# Role of ten‐eleven translocation proteins and 5‐hydroxymethylcytosine in hepatocellular carcinoma

**DOI:** 10.1111/cpr.12626

**Published:** 2019-04-29

**Authors:** Penghui Wang, Yunmeng Yan, Wei Yu, Hongyi Zhang

**Affiliations:** ^1^ Department of General Surgery Beijing Tiantan Hospital, Capital Medical University Beijing China; ^2^ Key Clinical Laboratory of Henan Province, Department of Clinical Laboratory The First Affiliated Hospital of Zhengzhou University Zhengzhou China

**Keywords:** 5‐hydroxymethylcytosine, epigenetic biomarkers, hepatocellular carcinoma, TET proteins, therapy and prognosis

## Abstract

In mammals, methylation of the 5th position of cytosine (5mC) seems to be a major epigenetic modification of DNA. This process can be reversed (resulting in cytosine) with high efficiency by dioxygenases of the ten‐eleven translocation (TET) family, which perform oxidation of 5mC to 5‐hydroxymethylcytosine (5hmC), 5‐formylcytosine and 5‐carboxylcytosine. It has been demonstrated that these 5mC oxidation derivatives are in a dynamic state and have pivotal regulatory functions. Here, we comprehensively summarized the recent research progress in the understanding of the physiological functions of the TET proteins and their mechanisms of regulation of DNA methylation and transcription. Among the three TET genes, TET1 and TET2 expression levels have frequently been shown to be low in hepatocellular carcinoma (HCC) tissues and received most attention. The modulation of TET1 also correlates with microRNAs in a post‐transcriptional regulatory process. Additionally, recent studies revealed that global genomic 5hmC levels are down‐regulated in HCC tissues and cell lines. Combined with the reported results, identification of 5hmC signatures in HCC tissues and in circulating cell‐free DNA will certainly contribute to early detection and should help to design therapeutic strategies against HCC. 5hmC might also be a novel prognostic biomarker of HCC. Thus, a detailed understanding of the molecular mechanisms resulting in the premalignant and aggressive transformation of TET proteins and cells with 5hmC disruption might help to develop novel epigenetic therapies for HCC.

## 1 INTRODUCTION

Hepatocellular carcinoma (HCC) ranks as the sixth most common cancer and the third leading cause of cancer‐associated deaths globally, with estimated 70 000 deaths annually.^1^, ^2^ The most significant risk factor of HCC is chronic fibrotic liver disease, mainly caused by hepatitis virus (B and C) infection and alcohol abuse.^2^ HCC is highly refractory to chemotherapy. Even after complete HCC tumour resection or ablation, approximately 70% of patients experience tumour recurrence within 5 years, which will continue to progress into incurable advanced‐stage disease.^3^, ^4^ Despite intensive research, the prognosis of HCC patients remains dismal, with an overall 5‐year survival rate <15%.^5^There is an urgent need for novel biomarkers to develop preventive strategies and therapeutic interventions based on an improved understanding of molecular hepatocarcinogenesis. It has been shown that both genetic and epigenetic alterations are crucial for the initiation of HCC, thus making epigenetics a promising and attractive field for identifying the subset of patients at a high risk of recurrence and with dismal survival outcomes.^6^, ^7^, ^8^, ^9^

In mammals, methylation of the fifth position of cytosine (5mC) in DNA has been one of the most broadly investigated epigenetic modifications and has critical functions in development and diseases.^10^, ^11^ 60%‐80% of CpG sites in the mammalian genome are modified by 5mC.^10^ Some of the well‐known functions of 5mC include regulation of genomic imprinting, X‐chromosome inactivation, inhibition transposable elements and participation in transcription.^12^

In the past decades, DNA methylation has been thought to be highly chemically and genetically stable.^13^ Nonetheless, mammalian 5mC can be reversed to its unmodified form in multiple ways. Firstly, blockage of the DNA methylation maintenance mechanism can cause the dilution of 5mC during DNA replication, a procedure referred to as passive demethylation.^13^, ^14^ Secondly, enzymes of ten‐eleven translocation (TET) family (TETs, including TET1, TET2 and TET3) can successively oxidize 5mC to 5hmC, 5‐formylcytosine (5fC) and 5‐carboxylcytosine (5caC). Furthermore, the excision of 5fC and 5caC mediated by thymine DNA glycosylase (TDG) followed by the base excision repair (BER) pathway will also result in demethylation.^15^, ^16^, ^17^ This process, the TET‐TDG pathway, referred to as active DNA demethylation, has received the most attention. Increasing evidence indicates that these 5mC oxidation products are not short‐term transient intermediates in an enzymatic pathway participating in DNA methylation. They can be steadily incorporated into the genomic DNA with high stability and can perform unique and significant regulatory functions in the mammalian genome.^18^, ^19^, ^20^, ^21^

Disruption of epigenetic patterns, including DNA methylation landscapes, is a hallmark of cancer. In this review, we summarize current advancements in the understanding of the machinery and functions of TET‐mediated DNA demethylation. Additionally, recent studies indicate that genomic 5hmC is globally down‐regulated in HCC tissues and cell lines. In combination with the existing scientific data, evaluation of 5hmC signatures in HCC tissues and in circulating cell‐free DNA should certainly contribute to early detection and help to devise therapeutic strategies against HCC. Furthermore, 5hmC might be a novel prognostic biomarker for HCC patients. Thus, precise modulation of DNA methylation landscapes, which is partly regulated by TET proteins, is crucial for HCC initiation and progression and offers a fundamental protection against malignant cellular transformation.

## 2 STRUCTURE AND FUNCTION OF TET PROTEINS IN RELATION TO THE REGULATION OF DNA METHYLATION PATTERNS

Three mammalian TET proteins have been identified so far, which catalyse the sequential oxidation of 5mC to 5hmC, 5fC and 5caC (collectively called oxidized 5mC).^22^, ^23^, ^24^ Furthermore, oxygen, Fe (II) and α‐ketoglutarate (α‐KG) are indispensable for the TET enzymes to perform the successive oxidation of 5mC and of its two intermediate oxidized derivatives, including 5hmC and 5fC. The final oxidized product is 5caC.^23^, ^25^

TET proteins are multi‐domain enzymes with the molecular mass ranging from 180 to 230 kDa. The core catalytic domain at the C‐terminus of all three TET proteins typically consists of a cysteine‐rich domain, a conserved double‐stranded β‐helix (DSBH) domain.^26^ Fe (II), α‐KG and 5mC are brought together by the DSBH domain for subsequent catalytic oxidization, whereas the cysteine‐rich domain surrounding the DSBH core makes the integrated catalytic core and TET DNA interaction stable. The TET DNA contact is not dependent on the methyl group, meaning that TET proteins accommodate various patterns of modified cytosine.^27^ Structural studies indicate that the core catalytic domain preferentially binds cytosines in a CpG‐dependent manner but does not interact with the surrounding DNA bases and shows little or no specificity for flanking DNA sequences.^27^, ^28^ Apart from their catalytic region, TET1 and TET3 share an N‐terminal CXXC zinc finger domain that can bind DNA.^29^, ^30^ The catalytic region of the C‐terminus alone can be targeted for relocation to the nucleus and implement the oxidization of 5mC.^22^, ^29^, ^31^

**Figure 1:**
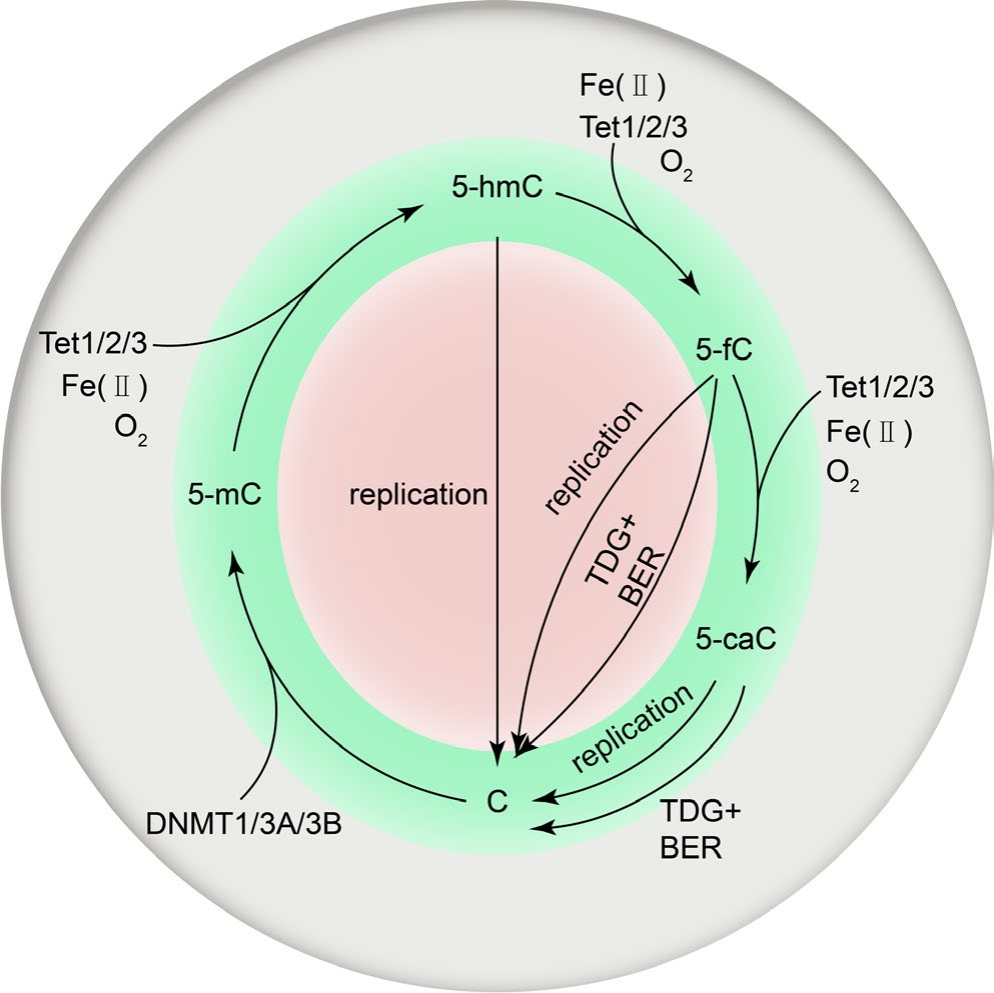
A schematic outline of the association between 5mC and 5hmC. 5mC generated from post‐duplicative transfer of the methyl group to cytosine via the catalysis by DNMTs, which utilize S‐adenosyl methionine (SAM) as a methyl donor. There are three known mammalian TET proteins at present, which catalyse the sequential oxidation of 5mC to 5hmC, 5fC and 5caC (collectively known as oxidized 5mC). Furthermore, oxygen, Fe(II) and α‐KG are indispensable for the TET enzymes to perform the successive oxidation of 5mC and of its two intermediate oxidized derivatives, 5hmC and 5fC. The final oxidized product is 5caC

TET proteins are obviously involved in the pathway of DNA demethylation.^26^, ^32^ Firstly, under the conditions where the maintenance methyltransferase DNMT1 is absent or prevented from accessing newly generated DNA, 5mC could be diluted during DNA replication, a process referred to as passive DNA demethylation. Secondly, 5hmC and probably other oxidized 5mC forms interact with maintenance methylation by inhibiting DNA binding with the UHRF1‐DNMT1 complex; thus, TET enzymes are highly likely to contribute to passive DNA demethylation.^33^, ^34^ Thirdly, TET proteins are implicated in active demethylation, an enzymatic process in which the bases of 5mC as well as their oxidized forms are replaced with unmethylated cytosines in a replication‐independent pattern. Even though multiple machineries have been proposed, the signalling cascade involving TDG‐mediated base excision and DNA BER is now considered the main driver of DNA demethylation.^18^ Remarkably, dysfunction of TET proteins does not always result in up‐regulation of 5mC due to aberrant DNA demethylation, indicating that oxidized intermediates of 5mC could serve as stable epigenetic biomarkers regardless of their functions in the DNA methylation process, probably by replacing methyl CpG‐binding proteins, by targeting specific proteins that interact with oxidized forms of 5mC or by changing the epigenetic modification of histones.^35^, ^36^ A schematic outline of the association between 5mC and 5hmC has been shown in Figure 1.

## 3 DISCOVERY AND DOWN‐REGULATION OF 5HMC

Recently, it was demonstrated that enzymatic oxidation of 5mC to 5hmC might serve as a common modification of DNA. Downstream erasure of 5hmC could actually be part of a sophisticated process of epigenetic gene regulation.^15^ 5hmC was first identified in the DNA of T‐even bacteriophages and detected in the genomic DNA of mammalian organs such as the brain and liver during the 1970s, but its functional significance was not appreciated comprehensively.^37^, ^38^ In 2009, the catalytic function of the TET proteins was unambiguously proven: conversion of 5mC to 5hmC, which has been rediscovered in substantial amounts in the murine brain, embryonic stem cells, Purkinje cells and granule cells.^22^, ^39^ 5hmC has been thought to perform an essential function in cellular differentiation and embryonic stem cells.^31^ It has been reported that genomic 5hmC content is reduced overall in solid carcinoma tissues including HCC.^18^, ^40^, ^41^, ^42^ Mutations and down‐regulation of TET genes could result in lower amounts of 5hmC in cancer tissues as compared to normal controls.^43^, ^44^, ^45^ Nonetheless, a full understanding of the exact molecular significance of 5hmC in HCC is still lacking.

## 4 POST‐TRANSCRIPTIONAL REGULATION OF TET1 BY MULTIPLE MICRORNAS

MiRNAs consist of a broad spectrum of small highly conserved noncoding RNAs that bind to the 3' untranslated regions (UTRs) of protein‐coding mRNAs to inhibit protein expression.^46^ Emerging evidence reveals that miRNAs can serve as tumour suppressors or oncogenic promoters, depending on the function of their target gene within a certain cell or tissue type.^47^, ^48^ Song et al have demonstrated that miR‐22 exerts its pro‐metastatic action by repressing anti‐metastatic miR‐200 via direct combining of TET family members, thus restraining miR‐200 promoter demethylation.^49^ This finding elucidates the relations among microRNAs, TET family members and DNA demethylation during the formation and progression of cancer.

Zhang et al have shown that TET1 was the direct target of miR‐29a through the 3′‐UTR of mRNA.^50^ Further evidence has revealed that the miR‐29 family modulates active DNA demethylation through direct targeting of TET1 in multiple solid cancers.^51^, ^52^ Lin et al have investigated the potential function of the negative feedback of miR‐29‐TET1 in HCC tumorigenesis and its potential mechanisms.^53^ Furthermore, miR‐29a overexpression up‐regulates DNA methylation of the suppressor of cytokine signalling 1 (OSCS1) promoter; this process has been implicated in HCC metastasis in vitro and in vivo. In terms of mechanism, miR‐29a down‐regulates anti‐metastatic OSCS1 by directly targeting the TET family, thus leading to repression of OSCS1 promoter demethylation. Finally, miR‐29a up‐regulation is associated with dismal clinical outcomes and with repression of TET‐SOCS1‐matrix metalloproteinase (MMP) 9 axis in HCC patients. These findings mean that miR‐29a is a crucial epigenetic regulator, facilitating HCC metastasis via the TET‐SOCS1‐MMP9 axis silencing.^54^

Aside from the miR‐29 family, there are other miRNAs participating in TET regulation in HCC. MiR‐494 is up‐regulated and involved in the silencing of several invasion‐suppressor miRNAs by direct targeting of TET1, thus resulting in vascular invasion of a tumour.^55^ Moreover, TET1 has been reported to be the direct target of miRNA‐191, whose aberrant overexpression reduces the expression of TET1 in intrahepatic cholangiocarcinoma. The decreased TET1 expression helps the methylated CpG‐rich segments at a promoter site of the *p53* gene to stay methylated, resulting in down‐regulation of *p53*, thereby disrupting the anti‐cancer function of *p53*. Overall, the up‐regulated miR‐191 is related to intrahepatic cholangiocarcinoma progression via the miR‐191/TET1/*p53* pathway.^56^ Furthermore, miR‐520b inhibits cell proliferation by reducing the expression of TET1.^57^ In this process, however, TET1 may act as an oncogene, and this notion contradicts results of a previous study. The paradoxical function has yet to be elucidated and might be partly attributed to a multitude of mechanisms of HCC aetiology and the complexity of clinical specimens.^57^

Increasing evidence has shown that TET1 expression can be modified by microRNAs, but TET1 can also modulate the miRNA expression pattern via its demethylation activity. Not only can TET1 be modulated by microRNAs, but TET1 can also induce several invasion‐suppressor microRNA genes through DNA demethylation in their proximal CpG locus.^49^, ^55^, ^58^ Hence, it is difficult but necessary to investigate the relevant regulatory machineries that orchestrate transcriptional and/or post‐transcriptional regulation during the initiation and progression of HCC.

## 5 THE EXPRESSION PATTERNS OF TET1 PROTEINS IN HCC

Reduced expression of TET1 has been demonstrated in a variety of malignant tumours, and this phenomenon is accompanied by a decrease in global 5hmC amounts and accumulation of ectopic DNA methylation.^18^, ^59^ Among the multiple types of cancer investigated, the function of TET family members in HCC has received much attention. These pieces of evidence indicate that the change of expression of TET proteins in tumour tissues plays a major part in hepatocarcinogenesis and tumour progression.

Multiple studies have revealed that the expression of TET1 proteins is significantly decreased in HCC tissues compared with normal noncancerous counterparts.^43^, ^53^, ^54^, ^59^, ^60^, ^61^, ^62^ Moreover, the mRNA levels of all three TET proteins are dramatically decreased in the adult liver when compared to foetal liver.^63^ Considering the function of TET in metastasis and cell adherence, Tao et al have found that TET1 silencing by siRNA can attenuate the inhibitory effect on cell migration caused by ALDOB overexpression.^64^ Likewise, Lin et al have demonstrated that TET1 inhibits cell proliferation by mediating a G1 arrest and suppression of cell migration and invasion in HepG2 and Huh7 cells. The effects of miR‐29 on apoptosis might be partly regulated by the modulation of TET1.^53^ In addition, miR‐29a down‐regulates 5hmC by suppressing TET family expression and the sequential epigenetic SOCS1 inactivation. Finally, dysfunction of SOCS1 triggers the up‐regulation of MMP9, which enhanced HCC cell proliferation, invasion and metastasis. Overall, miR‐29a regulates the HCC progression through the TET‐SOCS1‐MMP9 axis, which may represent a novel target for a therapeutic intervention in HCC progression.^54^

A knockdown of the TET1 gene can facilitate HCC cell invasion; this observation is consistent with some reports indicating that TET1 silencing increases mammary and prostatic cancer invasion and metastasis.^65^ Evidence has shown that miR‐494–induced HCC cell invasion can be abrogated by increased expression of TET1 because it could trigger several invasion‐suppressor miRNA genes by DNA methylation in their proximal CpG regions, implying that TET1 might function as a pivotal gene target in the regulation of the inhibitory effect of miR‐494 on HCC cell invasiveness.^55^ In intrahepatic cholangiocarcinoma, the decreased expression of TET1 can compromise the anti‐cancer potential of *p53*. Mechanistically, TET1 directly targets the transcriptional start site of the *p53* gene and promotes the expression of p53 by demethylating the CpG islands of the transcriptional start site within the *p53* gene.^56^

## 6 THE DISRUPTION OF TET2 OR TET3 IN HCC

Compared with TET1 disruption, somatic alterations and functions of TET2 and TET3 are comparatively rare in patients with HCC. Nevertheless, the expression and activity of proteins TET2 and TET3 proteins in the progression of HCC were recently investigated. Liu and colleagues have demonstrated that a decrease in 5hmC amounts is related to the progression of HCC via down‐regulation of the TET1 protein.^18^ In contrast, Gao and colleagues^62^ have evaluated nine HCC samples and demonstrated a decrease in TET1 and TET2 but not TET3 mRNA expression with concomitant down‐regulation of 5hmC. In line with its mRNA level, TET2 protein was then confirmed by Western blotting to be down‐regulated. Furthermore, it has been reported by Yang et al^43^ that the expression of all three TET proteins is dramatically and uniformly lower in HCC specimens when compared with their adjacent normal tissue samples. Liu et al have found a significant reduction in the TET2 level in 106 HCC samples when compared to their matched para‐tumour samples. They also demonstrated an insignificant decrease in TET1 and TET3 expression in a cohort containing 52 HCC patients. A decreased in TET2 and TET3 expression in HepG2 and Huh7 cell lines integrated with HBV DNA has also been observed.^61^

Additionally, Sajadian et al have demonstrated that the expression and activity of proteins TET2 and TET3 but not TET1 are impaired in HCC,^66^ thereby resulting in the down‐regulation of 5hmC. Furthermore, this study confirmed the specific action of 5‐azacytidine (5‐AZA): facilitation of a TET‐regulated generation of 5hmC, indicating that the availability of 5‐AZA in HCC cells will have multiple effects on different epigenetic targets. Further investigation has indicated that vitamin C increases active demethylation induced by 5‐AZA by enhancing the expression of TET2 and TET3. The combination of vitamin C and 5‐AZA decreases Snail gene expression and up‐regulates P21 with subsequent activation of GADD45B. The complex network of interactions among GADD45, P21, cyclin B1 and PCNA is the major mechanism causing cell cycle arrest in HCC.^67^

In summary, active demethylation that is mediated by TET proteins may also be an essential step for control over global methylation. Nevertheless, it remains unclear and hotly debated which TET proteins participate and their precise up‐ or down‐regulation and functions in the demethylation process during HCC tumorigenesis. The disruption of TET proteins offers a potential biological molecular machinery for a global reduction in the 5hmC amount in HCC. The detailed impact of disrupted TET activity on the progression and a potential therapeutic target of HCC need further research for confirmation. The expression patterns of TET proteins in HCC are summarized in Table 1.

**Table 1:** The expression patterns of TET proteins in HCC

Methods	Expression pattern	Year	Number of HCC samples	TET1 expression	TET2 expression	TET3 expression	Reference
Western blotting	Protein level	2013	20 pairs	Significantly decreased	Comparable expression	Comparable expression	59
IHC staining	Protein level	2015	25 pairs	Significantly decreased	Not mentioned	Not mentioned	53
RT‐PCR	mRNA level	2015	20 pairs	Significantly increased	Not mentioned	Not mentioned	57
RT‐PCR	mRNA level	2015	9 pairs	Comparable expression	Significantly decreased	Significantly decreased	66
RT‐PCR	mRNA level	2018	54 pairs	Comparable expression	significantly decreased	Comparable expression	61
RT‐PCR	mRNA level	2018	52 pairs	Comparable expression	Significantly decreased	Slightly decreased	61
RT‐PCR	mRNA level	2014	9 pairs	Significantly decreased	Significantly decreased	Comparable expression	62
RT‐PCR	mRNA level	2017	108 pairs	Significantly decreased	Significantly decreased	Significantly decreased	54
IHC staining	protein level	2017	323 pairs	significantly decreased	Significantly decreased	Significantly decreased	54
RT‐PCR	mRNA level	2013	3 pairs	Significantly decreased	Significantly decreased	Significantly decreased	43

Abbreviation(s): IHC, immunohistochemical; RT‐PCR, real‐time PCR.

## 7 ACTIVE DNA DEMETHYLATION IN HEPATIC PLURIPOTENCY AND DIFFERENTIATION

DNA methylation has been shown to have an important function in early development, whereby peaks of demethylation and re‐methylating have been found as part of a genome‐wide shaping of chromatin. The participation of 5mC in stem cell differentiation has been confirmed by multiple studies of enhanced differentiation after exposure to demethylating drugs.^68^ Further evidence originates from reprogramming experiments,^69^, ^70^, ^71^ including efforts to directly convert hepatic cell lineages by the expression of specific transcription factors.^72^, ^73^, ^74^ Hepatic stem/progenitor cell maintenance has been difficult to achieve; this problem is attributed to the confounding factors of other crucial mechanisms during the differentiation. Understanding the function of oxidization patterns of 5mC, and particularly 5hmC, in the control over liver progenitor cell differentiation will uncover molecular mechanisms of regenerative activity in liver tissue and novel therapeutic targets in HCC.

Given that 5mC, 5hmC and 5caC amounts are highly dynamic during early embryonic development, little is known about their precise functions in later processes of differentiation. Ancey et al have shown that 5hmC precedes the expression of promoter 1–dependent isomerides HNF4A, a primary transcription factor of hepatocyte differentiation. 5hmC and HNF4A expression from promoter 1 depends on TET dioxygenases. Moreover, the targeted connection of TET1 to the promoter 1 region is dependent on the liver pioneer factor FOXA2. Both TETs and FOXA2 are essential for the 5hmC‐associated conversion of HNF4A expression, indicating the early phase of bipotent liver progenitor differentiation.^75^ Consistently with a well‐known function in neural development,^76^, ^77^ 5hmC and TETs recently were found to participate in pivotal processes of terminal differentiation, for example monocyte‐to‐macrophage differentiation,^78^ specification of CD4^+^ T cells,^79^ colonocyte differentiation^80^ and cardiomyocyte development.^81^

Many works have proven that 5fC and 5caC can be specifically detected and removed from DNA by the TDG‐BER‐dependent pathway.^23^, ^82^ Transient accumulation of 5caC is suggestive of participation of active demethylation in lineage specification of neural stem cells.^83^ Furthermore, the TDG‐BER‐dependent pathway has been implicated in hepatic differentiation. By analysing the dynamics of the general expression levels of 5hmC and 5caC during differentiation of human pluripotent stem cells into hepatic endoderm, Lewis LC et al have shown that 5caC transiently accumulates during hepatic differentiation. The expression of 5caC increases gradually during specification of the foregut, fluctuates at the phase of hepatic endoderm commitment and diminishes within differentiated cells simultaneously with the initial expression of AFP, a biomarker of committed hepatic progenitors.^84^ In addition, they observed accumulation of 5caC in the promoter regions of multiple genes expressed during hepatic specification in differentiation phases concurrently with the onset of their expression. Thus, the transitory accumulation of 5caC is a global pattern of two different categories (neural/glial and endodermal/hepatic) of cellular differentiation. This result means the active demethylation by DNA repair might represent a common machinery for resetting the 5mC patterns during terminal differentiation of somatic cells in mammals.

Besides, Ivanov et al have revealed that the adult human liver contains a variable but significantly higher level of 5hmC compared to foetal liver specimens. Prominent differences in both general 5hmC content and genomic distribution between adult and foetal livers have also been confirmed.^63^ Genomic distribution according to next‐generation sequencing has revealed 5hmC dramatically accumulates in the coding regions of actively transcribed genes that mainly participate in metabolic and catabolic processes. These differences indicate that 5hmC performs a crucial function in the development and maturation of the human liver and might be a pivotal indicator of HCC initiation.

## 8 HCC IS ASSOCIATED WITH DECREASED 5HMC LEVELS

It has been demonstrated that the global 5hmC content is reduced overall in cancer specimens.^18^, ^40^, ^43^, ^85^ The dramatic decrease in 5hmC amounts in several types of solid cancers points to global epigenetic and transcriptomic perturbations during malignant transformation, concomitant with dysfunction and/or aberrant activity of the TET proteins.^18^

In genomic DNA from the human adult liver, the level of 5hmC is about 1% of the total cytosine content, indicating that this base has a crucial function in the regulation of gene expression.^63^ Multiple studies have revealed global reduction of 5hmC amount in HCC tissues and cell lines.^42^, ^54^, ^59^, ^60^, ^61^, ^62^, ^86^, ^87^, ^88^ Chen et al have confirmed that the expression level of 5hmC is higher in weakly metastatic HCC cell lines (HepG2 and SMMC‐7721) and lower in highly metastatic HCC cell lines (MHCC97H and HCCLM3) as compared to the normal liver cell line L‐02.^54^ Of note, Liu et al have demonstrated that the global amount of 5hmC is dramatically lower in the genome of HCC cell lines transfected with HBV DNA, in comparison with their parental cells.^61^ Indeed, it was reported recently that the expression of both TET1 and global 5hmC levels is lower in proliferating cells in culture just as in proliferating hepatocytes during liver regeneration after partial hepatectomy.^89^, ^90^ Taken together, these findings suggest that HCC tissues and tumour cells have globally lower 5hmC amounts in genomic DNA, and HBV infection may result in a more aggressive abnormality. Increasing evidence has suggested 5hmC loss is an epigenetic indicator of HCC and is tightly and broadly correlated with tumour progression.

## 9 ASSOCIATION OF 5HMC WITH CLINICAL AND BIOCHEMICAL CHARACTERISTICS OF HCC PATIENTS

Evidence has shown that reduced 5hmC amounts in genomic DNA may correlate with HCC development. Therefore, the association of 5hmC content with clinical characteristics in HCC has also been extensively investigated. Chen et al have demonstrated that the 5hmC content is dramatically associated with HCC TNM stage, and a reduced 5hmC amount correlates with tumour progression.^54^ Likewise, Chen et al have discovered that the 5hmC staining score is inversely associated with the TNM stage. Particularly, 5hmC levels can be utilized to discriminate HCC patients at TNM stage I from stage II‐III.^42^ These results are in agreement with the findings that a global reduction in 5hmC contents in HCC genomic DNA takes place in the very early stage (BCLC stage 0) and correlates with HBV infection.^61^

In another study, Liu et al have evaluated the clinical relevance of 5hmC expression in two separated cohorts containing 318 patients and 328 patients who underwent surgical resection.^86^ In the training group, the 5hmC expression was associated with sex and the AFP levels. In the validation group, 5hmC expression was associated with sex, age, the AFP level, tumour number and TNM stage. Furthermore, Shen et al have found that 5hmC is related to tumour size. According to the subgroup analyses depending on tumour size, HCC subjects with a tumour size ≤5.0 cm have higher 5hmC content and lower levels of fasting plasma aspartate aminotransferase, the ratio of alanine aminotransferase to aspartate aminotransferase, c‐glutamyl transferase and AFP in comparison with the patients with tumour size ≥5 cm.^87^

These findings suggest that 5hmC is associated with the damage liver function. Therefore, application of 5hmC in combination with routine testing of liver functional parameters may serve as a novel biomarker for early detection of HCC.

## 10 THE POTENTIAL OF 5HMC AS A DIAGNOSTIC BIOMARKER OF HCC

Chronic liver inflammation has always preceded the majority of HCC cases. Ectopic epigenetic alterations increased gradually in the chronically inflamed liver and might facilitate the progression of HCC. Chronic inflammation does not alter global levels of DNA methylation in specific genetically engineered mice but decreases of global 5hmC amounts and up‐regulates TET1 expression.^88^ Furthermore, chronic liver inflammation leads to hypermethylation of specific CpG islands, which may influence both non‐hepatocytes and hepatocytes in the liver. In particular, methylation of specific CpG islands is higher during the progression from chronic hepatitis to cirrhosis and to HCC, leading to the inhibition of some tumour suppressor genes. These alterations indicate that 5hmC may serve as a potential diagnostic biomarker of a higher regenerative activity and of a late precancerous stage in the chronically inflamed liver.

5hmC is a major mammalian DNA epigenetic mark that has been associated with global gene regulation and tumorigenesis. Via a sensitive chemical labelling‐dependent low‐input shotgun sequencing approach, Song et al have evaluated the diagnostic potential of 5hmC in circulating cell‐free DNA.^91^ They sequenced cell‐free 5hmC from HCC patients and discovered profound patterns that could be utilized to separate HCC cases from each other and healthy subjects and predict TNM stage with high accuracy. Similarly, Li et al have investigated disease specificity of a particular classifier in patients with HCC. The 5hmC biomarker originating from plasma cell‐free DNA is highly predictive of HCC, with a 44% call rate.^92^ Furthermore, after following up four HCC patients who underwent surgical resection, they confirmed that the cell‐free 5hmC sequencing offers an opportunity to detect HCC, as well as supervise treatment and recurrence of HCC.

Evidence has shown that cell‐free 5hmC profiling might be employed not only to predict HCC but also to identify tumour stage. By receiver‐operating curve analysis, Chen et al further assessed the possibility of 5hmC presence in genomic DNA of HCC tissues as an indicator for early detection of HCC. Remarkably, 5hmC showed high efficiency of HCC diagnosis, with 91.7% sensitivity and 98.6% specificity (area under the curve = 0.969).^42^

Collectively, genome‐wide signatures of 5hmC in circulating cell‐free DNA and genomic DNA of HCC specimens have high predictive value in terms of HCC. 5hmC disturbance patterns could serve as a biomarker for the early detection of HCC. Nevertheless, the precise biological importance of 5hmC depletion in HCC remains to be elucidated.

## 11 CLINICAL IMPLICATIONS OF DECREASED 5HMC LEVELS IN HCC PROGNOSIS AND THERAPY

Multiple studies have revealed that a decrease in 5hmC levels in HCC tissues correlates with tumour stage and poor prognosis.^42^, ^54^, ^59^, ^61^, ^86^, ^87^ Subjects with lower 5hmC content in HCC specimens experience shorter overall survival and disease‐free survival. Furthermore, the reduced level of 5hmC in HCC and non‐tumour tissues correlates with early tumour recurrence after surgical resection.

Available data on 5hmC and IDH2 expression alterations in HCC revealed that low 5hmC and IDH2 expression correlates with aggressive properties of HCC. Reduced 5hmC or IDH2 amounts individually and combined 5hmC and IDH2 expression correlates with shorter overall survival and higher cumulative recurrence rates. Multiple analyses suggest that 5hmC or IDH2 and 5hmC + IDH2 are independent predictive factors for overall survival and disease‐free survival, thereby confirming that 5hmC may be a biological biomarker with high prognostic and predictive value.^86^

In addition, ALDOB and multiple miRNAs, some of which are up‐regulated in HCC, have been demonstrated to directly target TET proteins.^53^, ^54^, ^55^, ^57^, ^64^ Decreased expression of TET proteins and lower 5hmC levels are general hallmarks of multiple cancer types, including HCC.^18^ These pieces of evidence imply that miRNA repression or substitution might offer us a novel therapeutic approach to inhibit HCC invasion and distant metastasis. In particular, miR‐29a promotes a metastatic phenotype and its up‐regulation correlates with dismal clinical outcomes among HCC patients. Additionally, miR‐29a could be a useful indicator for evaluation of metastatic patterns of HCC. This notion indicates that miR‐29a repression by specific miR‐29a inhibitors in highly metastatic cells decreases the metastatic potential as well as increase the expression of TET proteins and SOCS1; this phenomenon represents a novel scheme for a therapeutic intervention that inhibits HCC metastatic phenotypes. Chuang et al have confirmed the viewpoint that repression of miR‐494 might be a promising method for sustaining sufficient expression of the TET proteins and of several invasion‐suppressor microRNAs as an innovative therapeutic method for inhibiting HCC vascular invasion and recurrence.^55^

Furthermore, up‐regulation of 5hmC by means of compounds that potentiate or increase TET function is expected to have extensive applications in the treatment of HCC, especially because it has been demonstrated that TET activity is impaired in various cancers. Vitamin C has also been shown to enhance TET activity in HCC cells, most likely by acting as a cofactor.^67^, ^93^, ^94^, ^95^ As for the mechanism, vitamin C directly interacts with the catalytic domain of TET proteins to increase their enzymatic activity.^94^, ^95^ It might be an effective compound for the modulation of TET activity in vivo.

**Figure 2:**
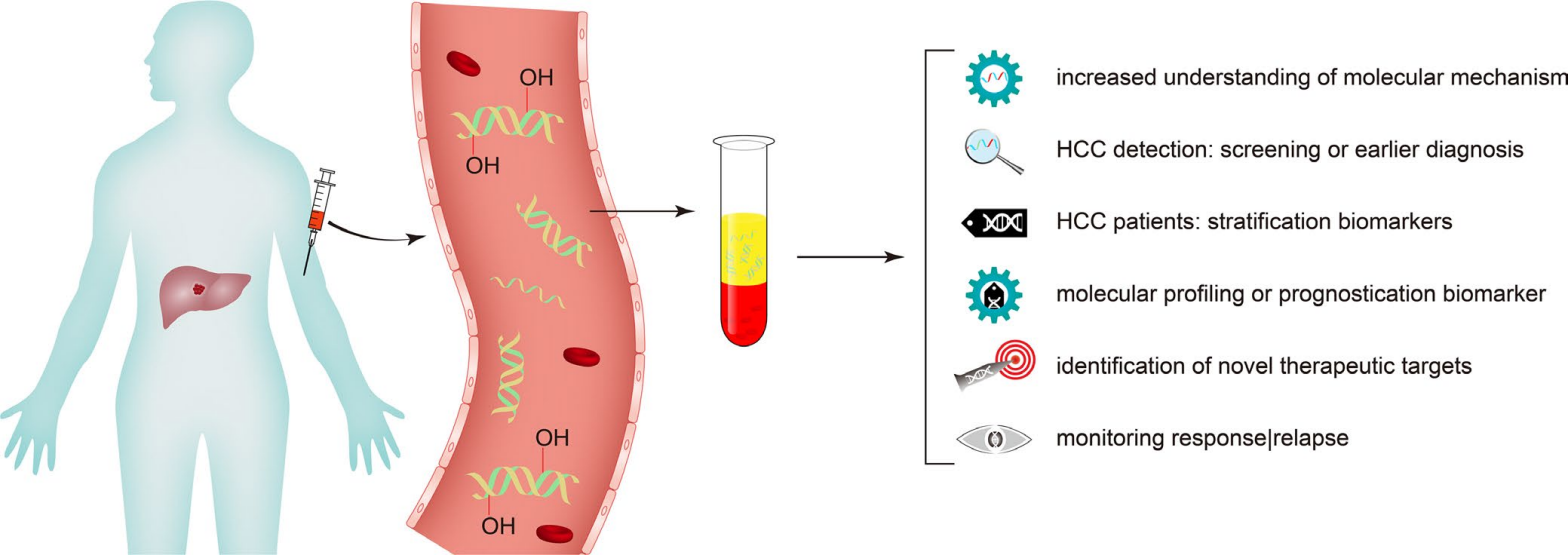
Applications of 5hmC in circulating cell‐free DNA in the course of HCC management. Here, we present a schematic diagram for early detection of HCC by means of cell‐free DNA 5hmC. Proof‐of‐principle results indicate that cell‐free DNA 5hmC signatures are useful for early detection of HCC and evaluation of tumour stage. Large‐scale clinical trials are necessary to fully validate the feasibility and to understand potential limitations of this approach. Cell‐free DNA 5hmC constitutes a novel dimension of information for liquid biopsy‐based diagnosis and prognosis. Collectively, levels of 5hmC in cell‐free DNA can be estimated and can contribute to (a) increased understanding of molecular mechanisms; (b) HCC detection: screening or earlier diagnosis; (c) HCC patients: stratification biomarkers; (d) molecular profiling or prognostication biomarker, and (e) identification of novel therapeutic targets and monitoring response and relapse

A schematic photograph of applications of 5hmC in circulating cell‐free DNA in the course of HCC management is present in Figure 2.

## 12 CONCLUSIONS

Since their discovery, much progress has been made in our basic and mechanistic understanding of TET protein participation in the control of DNA methylation and demethylation, embryonic development, and normal and malignant cellular transformation.^23^, ^35^ By regulating sequential oxidation of 5mC, TET proteins may facilitate DNA methylation in concert with DNA repair enzymes. Extensive studies have proved that TET proteins and DNA are involved in multiple processes of differentiation and cancer progression. For example, the enzymatic activity of TET proteins performs crucial function in induced pluripotent stem cell formation,^96^, ^97^ smooth cell differentiation, and hepatic pluripotency and differentiation.^75^, ^84^, ^98^ Furthermore, TET2 participates in the modulation of cytokine expression during an initial and T cell–regulated immune response. Regarding myeloid cell‐specific deletion, it has been demonstrated that TET2‐deficient cells fail to efficiently down‐regulate proinflammatory cytokines after LPS‐mediated stimulation in a histone deacetylase‐dependent manner.^99^ TET2 deficiency induced in CD2^+^cells correlates with abnormal cytokine production and enhances autoimmunity in mouse models of human multiple sclerosis.^100^ Recently, it was reported that mutations in TP53, IDH1, IDH2, DNMT3A, TET2 and spliceosome genes dramatically increase the risk of developing acute myeloid leukaemia.^101^ Nevertheless, the basic and underlying molecular mechanism remains to be fully elucidated: How does TET2 deletion promote malignant transformation? How can researchers optimize the potential practical value of TET2 alteration or modification for improving early detection of cancer and for immunotherapies?

Recent studies suggest that the global genomic 5hmC amount is reduced in HCC tissues and cell lines. The mechanisms responsible for such a reduction include (a) the reduction of 5mC content; (b) the reduced expression levels of TET enzymes and down‐regulation of α‐KG, a cofactor of TET enzymes; (c) uncoordinated expression of DNA methylation‐associated enzymes; and (d) HBV infection might also participate indirectly.^61^ Additionally, studies suggest that a decrease in 5hmC content correlates with the poor prognosis of HCC.

The genome‐wide epigenetics‐based technology continues to hold tremendous promise for both deepening our understanding of HCC progression at the molecular biological level and for the development of an effective, minimally invasive diagnostic strategy with direct clinical application.^61^, ^92^, ^102^ Not only are these epigenetic profiles strongly disturbed during the early stages of HCC progression, but increasing evidence also indicates that these are unique to specific cancer types. These data are suggestive of a potential application to the stratification of patients into optimal downstream therapeutic regimes. Linking these stratification schemes with joint testing of relevant clinical characteristics will be greatly useful. In contrast to genetic mutations, which were encoded in the DNA sequence, epigenetic alterations are potentially reversible. Therapeutic interventions against this early abnormality in patients with hematopoietic cancers by means of demethylating agents (decitabine and azacytidine) have not yielded conclusive results.^103^, ^104^, ^105^, ^106^ Although some studies suggest that TET2 mutations are associated with a better clinical response, such a treatment does not provide a survival benefit. Nevertheless, disruption of TET2 enhances the therapeutic efficacy of CD19‐targeted T cells, leading to deep molecular remission in a 78‐year‐old patient with chronic lymphocytic leukaemia.^107^ Thus, the development of refined drugs that reverse normal epigenetic signatures is greatly attractive in the field of cancer research, because such drugs have substantial clinical applications as effective therapies in combination treatments. Connecting genome‐wide epigenetics‐based agents with recent developments in CRISPR‐Cas9 technologies and expansion and persistence of CAR T cells will strongly contribute to future research into cancer progression and to identification of innovative cancer therapies.

## CONFLICTS OF INTEREST

No potential conflicts of interest existed.
